# Supplementary material for: Antibiotic dispensing practices and antimicrobial stewardship gaps in community pharmacies in Kakamega County, Kenya
Source: PLoS One. 2026 May 15;21(5):e0342353. doi: 10.1371/journal.pone.0342353 (PMC13178857; doi:10.1371/journal.pone.0342353)
Supplement: S1 Table — Summary of reported reasons for dispensing incomplete antibiotic courses across community pharmacies in Kakamega County, Kenya. (DOCX) [file pone.0342353.s001.docx]

**Supplementary Table S1. Reasons for partial antibiotic course dispensing (n = 33)**

Distribution of stated reasons among 33 partial-course events, including financial constraint, patient preference, and other contributing factors.

| **Reason** | **N** | **% of Partial-course events** |
| --- | --- | --- |
| Financial constraint | 15 | 45.5% |
| Patient preference for smaller quantity | 6 | 18.2% |
| Stock shortage | 4 | 12.1% |
| Adverse reaction or intolerance | 3 | 9.1% |
| Other / unspecified | 5 | 15.1% |

Notes
Percentages are calculated as the proportion of all partial-course dispensing events (n = 33).
