# Supplementary material for: Antibiotic dispensing practices and antimicrobial stewardship gaps in community pharmacies in Kakamega County, Kenya
Source: PLoS One. 2026 May 15;21(5):e0342353. doi: 10.1371/journal.pone.0342353 (PMC13178857; doi:10.1371/journal.pone.0342353)
Supplement: Attachment — Submitted filename: Response to Reviewers.docx [file pone.0342353.s004.docx]

Dear Editors,

Thank you for the opportunity to revise and resubmit our manuscript entitled **“Antibiotic dispensing practices and antimicrobial stewardship gaps in community pharmacies in Kakamega County, Kenya.”** We are grateful to the editor and reviewers for their constructive and insightful comments, which have helped us to substantially strengthen the manuscript.

We have carefully addressed all editorial and reviewer comments and provide a detailed, point-by-point response below. All changes have been incorporated into the revised manuscript.

**Editorial Comments**

**1. PLOS ONE formatting requirements**

We have revised the manuscript to fully comply with PLOS ONE formatting guidelines, including structure, section headings, and file naming conventions. The manuscript has been aligned with the PLOS ONE formatting templates provided.

**2. Inclusivity in global research questionnaire**

A completed copy of the PLOS ONE inclusivity in global research questionnaire has been included as Supporting Information (**S2 File**), and this is referenced in the Methods section of the manuscript.

**3. Ethics statement placement**

The ethics statement has been reviewed and is now included exclusively within the Methods section. It has been removed from any other sections to comply with journal requirements.

**4. Funding information consistency**

The Funding statement has been reviewed and harmonised across all submission fields. All funding sources are now consistently reported in both the manuscript and submission system. The funding statement now reads, ‘This work was supported by the Commonwealth Partnerships for Antimicrobial Stewardship (CwPAMS), funded by the UK Department of Health and Social Care (DHSC) through the Fleming Fund [project reference: CwPAMS 2.5 A.01], and by Cambridge Global Health Partnerships (CGHP). The funders had no role in study design, data collection and analysis, decision to publish, or preparation of the manuscript.’

**5. Supporting Information captions**

Captions for all Supporting Information files have been added at the end of the manuscript, and in-text citations have been updated to ensure consistency with PLOS ONE guidelines.

**6. Data availability statement**

The Data Availability statement has been revised to clarify that the anonymised dataset will be made publicly available in Figshare without restriction upon publication. Data are provided as Supporting Information for peer review.

**7. Reviewer-suggested citations**

We have reviewed all suggested citations and included relevant references where appropriate. No irrelevant or non-essential citations were added.

**8. Reference list accuracy**

The reference list has been carefully reviewed and updated to ensure accuracy, completeness, and consistency with in-text citations. No retracted articles are cited.

**Reviewer #1**

**Comment 1: Update AMR mortality data**

We have updated the Background section to reflect the most comprehensive available estimates, including both direct (1.27 million) and associated (4.95 million) AMR-related deaths globally in 2019, based on the latest global analysis.

**Comment 2: Expand conclusions beyond governance gaps**

The Conclusion has been revised to incorporate additional key findings, including:

- Substantial use of broad-spectrum antibiotics across multiple indications
- Financial barriers to completing antibiotic courses
- The central role of pharmacists in antibiotic selection

This provides a more comprehensive summary of the study’s implications.

**Reviewer #2**

**Comment 1: General feedback**

We thank the reviewer for their positive assessment of the manuscript.

**Comment 2: Study design clarification**

The study design has been revised from “prospective descriptive cross-sectional” to **“cross-sectional study”**, consistent with STROBE guidelines and the analytical nature of the study.

**Comment 3: Sampling justification**

We have clarified that all eligible dispensing events during the study period were included consecutively to minimise selection bias. We have also stated that a formal sample size calculation was not performed due to the observational design and defined study period.

**Comment 4: Clarification of “both” dispensing source**

We have clarified that cases classified as “both” refer to instances where a prescription was presented but additional antibiotics were requested and supplied without prescription.

**Comment 5: Multiple antibiotics per prescription**

We have added detail to the Results section specifying that 52 (10.3%) dispensing events involved more than one antibiotic, most commonly involving two antibiotics, with very few involving three or more.

**Comment 6: Inclusion of AWaRe classification in results**

We have incorporated the WHO AWaRe classification into the Results section, noting that the majority of antibiotics dispensed belonged to the Access and Watch categories, with frequent use of Watch antibiotics such as macrolides and fluoroquinolones. This has also been further discussed in the Discussion section.

**Additional Revisions**

In addition to the points raised by reviewers and editors, we have undertaken further refinements to improve clarity and internal consistency:

- **Table 2 has been fully revised** to ensure accurate, dataset-derived classification of antibiotics by clinical indication using consistent antibiotic class groupings.
- The **Results and Discussion sections have been updated** to ensure complete alignment with the revised table and underlying dataset.
- Minor language edits have been made throughout to improve clarity, precision, and readability.

We believe these revisions have significantly strengthened the manuscript and addressed all concerns raised during the review process. We are grateful for the opportunity to revise our work and hope it is now suitable for publication in PLOS ONE.

Thank you for your consideration.

Yours sincerely,
Eleanor Turnbull-Jones
(on behalf of all authors)
